# Poor prognostic role of the pretreatment platelet counts in colorectal cancer: A meta-analysis

**DOI:** 10.1097/MD.0000000000010831

**Published:** 2018-06-18

**Authors:** Xu-Dong Rao, Hua Zhang, Zheng-Shui Xu, Hua Cheng, Wei Shen, Xin-Ping Wang

**Affiliations:** a Department of General Surgery, GuangRen Hospital of Xi’an Jiaotong University, Xi’an No. 4 Hospital, Xi’an, Shaanxi; b Department of Breast Surgery, The Forth Affiliated Hospital of Nanchang University, Nanchang, Jiangxi; c Department of General Surgery, The Second Affiliated Hospital of Nanchang University, Nanchang, Jiangxi, China.

**Keywords:** colorectal cancer, meta-analysis, platelet count, survival

## Abstract

**Background:**

Recently, a wide variety of studies have suggested that elevated platelet counts are associated with survival in patients with colorectal cancer. On one hand several studies suggest a negative connection in colorectal cancer patients with pre-operative thrombocytosis, on the other hand other studies contradicts this. However, it remains unknown whether elevated platelet counts are associated with survival in colorectal cancer patients. We therefore conducted this meta-analysis to evaluate the prognostic role of platelet counts in colorectal cancer.

**Methods:**

PubMed, Embase, and the Cochrane Library databases were searched from their inception to October 15, 2016 to identify relevant studies that have explored the prognostic role of platelet counts in colorectal cancer. Studies that examined the association between platelet counts and prognoses in colorectal cancer and that provided a hazard ratio (HR) and 95% confidence interval (CI) for overall survival (OS) and/or disease-free survival (DFS) were included.

**Results:**

This meta-analysis included 9 retrospective cohort studies involving 3413 patients with colorectal cancer. OS was shorter in patients with elevated platelet counts than in patients with normal counts (HR 2.11, 95% CI: 1.68–2.65). For DFS, an elevated platelet count was also a poor predictor (HR 2.51, 95% CI: 1.84–3.43).

**Conclusion:**

In this meta-analysis, we suggest that an elevated platelet count is a negative predictor of survival in both primary colorectal cancer and resectable colorectal liver metastases.

## Abstract 2

Supplemental Digital Content is available in the text

## 1 Introduction

Colorectal cancer remains one of the most common malignancies and is a leading cause of cancer-related deaths around the world.^[1]^ Pretreatment imaging and tests for tumor-associated antigens remain the main methods used to evaluate prognoses in colorectal cancer patients, and no clear advances or new methods have presented themselves. We expect that novel markers can be identified that will be useful for predicting prognoses in colorectal cancer patients before treatment begins. Recently, a large number of studies have shown that thrombocytosis is associated with the development and progression of cancer.^[2–8]^ Thrombocytosis may therefore provide a new method for evaluating prognoses in colorectal cancer.^[1,2]^

In adults, platelet counts normally range from 100 to 300 × 10^9^/L. Under an activation situation, platelets can release granules containing a variety of contents that can both inhibit and stimulate plasmatic coagulation, angiogenesis immunosurveillance, or neoplasm growth. Platelets can therefore play important roles in several hallmarks of tumor pathophysiology, including immune escape in disseminated malignant cells, neoplasm growth, and metastasis.^[5,9–11]^ Similarly, the results of a growing number of clinical studies have indicated that platelet counts are associated with overall survival (OS) and disease-free survival (DFS) in colorectal cancer,^[8,12–14]^ but this claim remains controversial; some studies indicate that preoperative thrombocytosis is a poor predictor of survival in colorectal cancer patients, while others suggest that there is no correlation between preoperative thrombocytosis and the survival in colorectal cancer patients.^[14–17]^

Many meta-analyses have previously been performed to explore the prognostic role of an elevated platelet count as a predictor of survival in various cancers, such as hepatocellular carcinoma, renal cell carcinoma, lung cancer, and gastric cancer.^[18–21]^ Therefore, we sought to determine whether an elevated platelet count could be used to predict OS and DFS in colorectal cancer. We performed this meta-analysis to provide a new method for evaluating survival in colorectal cancer.

## 2 Materials and methods

All analyses were based on previous published studies, thus no ethical approval and patient consent are required.

### 2.1 Literature search and inclusion criteria

**Figure 1:**
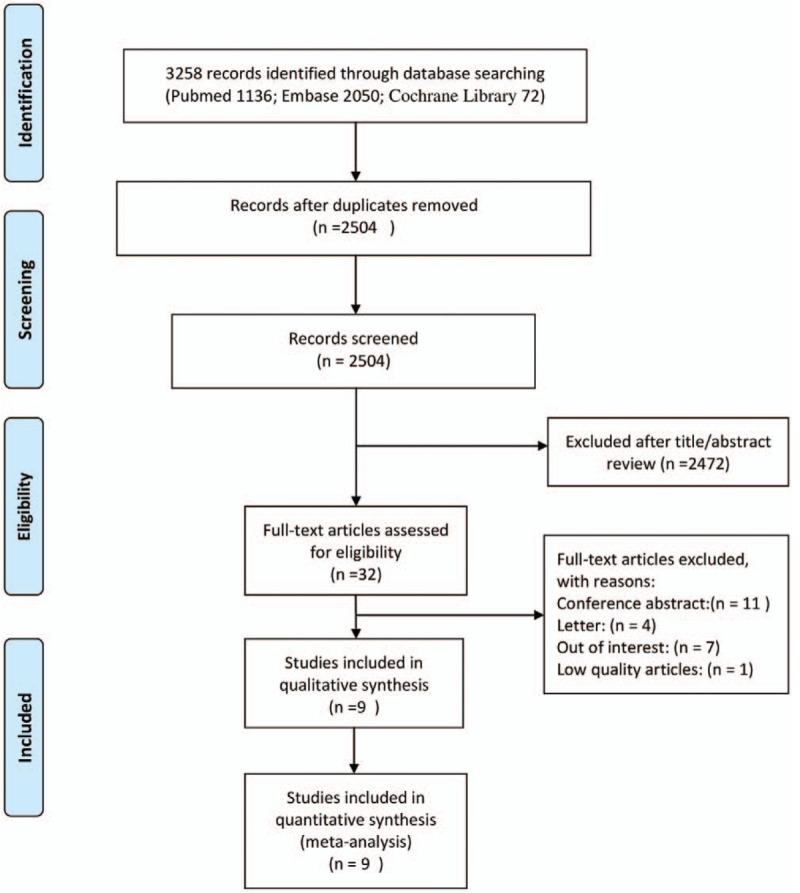
Diagram of study selection process.

PubMed, Embase, and the Cochrane Library databases were searched from their inception to October 15, 2016 to identify relevant studies that explored the prognostic role of platelet counts in colorectal cancer. No language restrictions were applied. We performed a search of titles and abstracts using the following terms: (“thrombocytosis” OR “thrombocythemia” OR platelet^∗^) and (“colorectal” OR “colorectum” OR “colon” OR “Rectum” OR “Rectal” OR “large intestine”) AND (adenocarcinoma^∗^ OR tumor^∗^ OR tumor^∗^ OR neoplasm^∗^ OR carcinoma^∗^ OR cancer^∗^ OR “malignant”). A MeSH/Emtree search for “blood platelets, colorectal cancer, rectal neoplasm, and colonic neoplasms” was also performed. The complete process that was used to search these 3 databases is shown in Appendix 1. Three reviewers independently performed the literature retrieval protocol according to previously chosen eligibility criteria. Any contradiction or inconsistency was resolved through iteration, discussion, and consensus. Moreover, we checked the references of all of the included studies and related reviews to retrieve any additional studies that should be included. The selection process used to identify the articles is shown in Fig. 1.

We only included studies in which patients with colorectal cancer underwent elective surgery R0 resection. The primary outcomes were OS and DFS, and we required that the studies provide a hazard ratio (HR) and 95% confidence interval (CI). The blood samples must have been obtained before the operation was performed. If not, the study was excluded. The detailed inclusion and exclusion criteria that were used in this study are presented (Table 1).

**Table 1:**
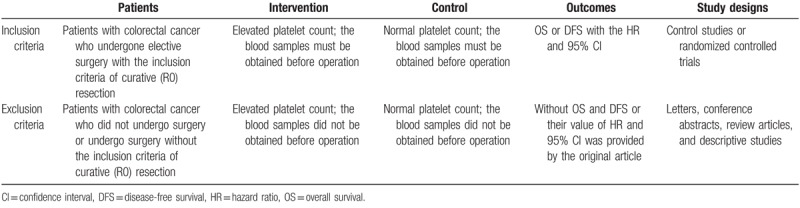
The detailed inclusion and exclusion criteria.

### 2.2 Data collection and assessment of methodological quality

All data were collected from the included studies using the Patients–Intervention–Control–Outcomes Study designs form. The following relevant information was extracted into our predesigned table: Patients (P): country, number, age, type of colorectal cancer, whether curative (R) resection was achieved.

Intervention (I): the cut-off value, time at which blood samples were obtained. Control (C): the cut-off value, time at which blood samples were obtained. Outcomes (O): the definition of and data obtained for OS and DFS. Study designs (S): the details used for study selection, comparisons made across data and outcomes, and follow-up. The same investigator assessed the quality of each study using the Newcastle–Ottawa Scale (NOS).^[22]^ Each study with NOS scores ≥6 was regarded as a high-quality study, while studies with NOS scores <6 were regarded as low-quality studies.

### 2.3 Statistical analysis

Meta-analysis inverse variance HR was performed for the primary outcome, HR and 95% CI values were used to estimate pooled outcomes.^[20]^ When considering potential difference between included studies, pooled outcomes were calculated by conservatively using a random-effects model. In addition, the I^2^ statistic was used to evaluate heterogeneity in the pooled outcomes. An I^2^ that was not <50% suggested the presence of significantly heterogeneity among the included studies. No significant heterogeneity was found among the included studies. Moreover, we performed subgroup analyses of the end-points of interest to determine whether there was potential heterogeneity among the included studies according to sample size, cut-off values, and type of colorectal cancer (e.g., primary colorectal or resectable colorectal liver metastases). Finally, a sensitivity analysis was performed using the “metainf” STATA command to validate the credibility of the pooled outcomes. The method of removing any study one at a time was applied. Because 2 of the pooled outcomes included fewer than 10 trials each, publication bias was not evaluated. A bilateral *P* < .05 was considered to indicate a significant difference. RevMan (version 5.2; Cochrane Library) and Stata 12.0 (StataCorp LP, College Station, Texas) were used to perform all statistical analyses.

## 3 Results

A total of 3258 records were identified through the search of the PubMed, Embase, and Cochrane Library databases. After we removed duplications and irrelevant studies, 32 potentially eligible studies were identified. After we scanned these 32 studies as full-text articles, 11 studies remained, and of these, 2 studies^[5,23]^ reported the same outcomes with overlapping study period and the 1 study^[11]^ was excluded because of low NOS scores. Finally, this meta-analysis included 9 retrospective cohort studies comprising 3413 patients with colorectal cancer.^[5,10,15,24–29]^

### 3.1 Characteristics of the included studies

All 9 studies were published in English between 2005 and 2016. Because one of the studies reported 2 trials that included primary colorectal and resectable colorectal liver metastases, a total of 10 trials were included in the 9 studies. In 6 out of the 10 trials,^[5,15,25,26,28,29]^ survival was reported for primary colorectal cancer. In the remaining 4 trials,^[5,10,24,27]^ survival was reported for resectable colorectal liver metastases. The characteristics of all of the included studies are presented (Table 2), and the analysis of the quality of all of the included studies is presented (Table 3).

**Table 2:**
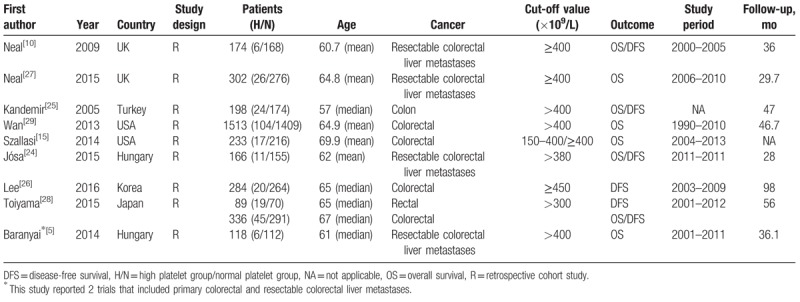
The characteristics of included studies.

**Table 3:**
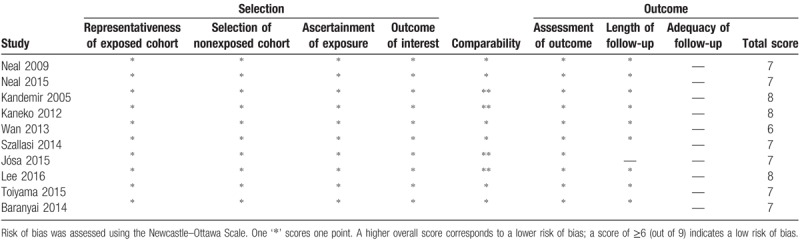
Risk of bias assessment of the included studies.

### 3.2 Outcomes

**Figure 2:**
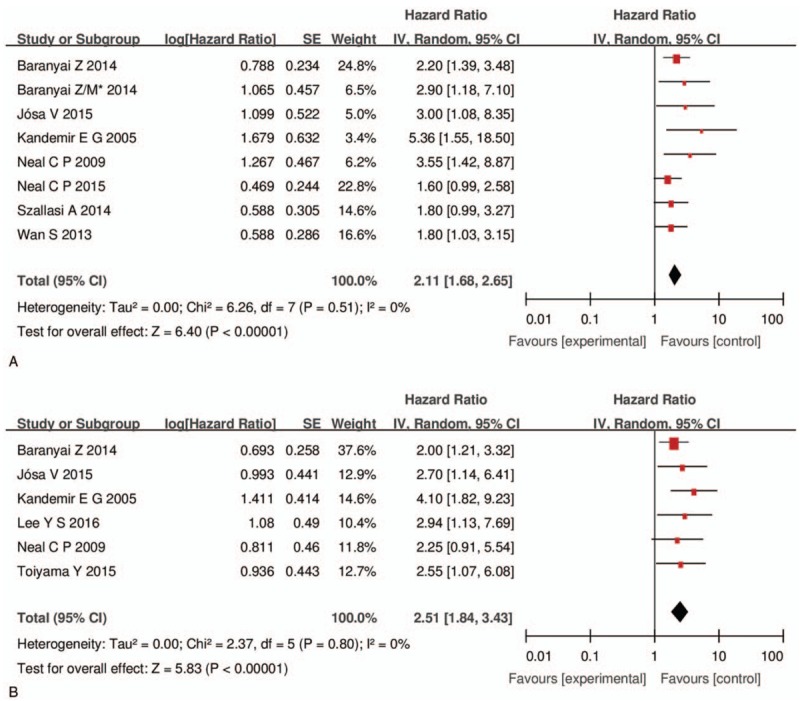
Forest plots of including studies evaluating hazard ratios of overall survival (A) and disease-free survival (B). CI = confidence interval, MH = Mantel–Haenszel, SE = standard error.

**Figure 3:**
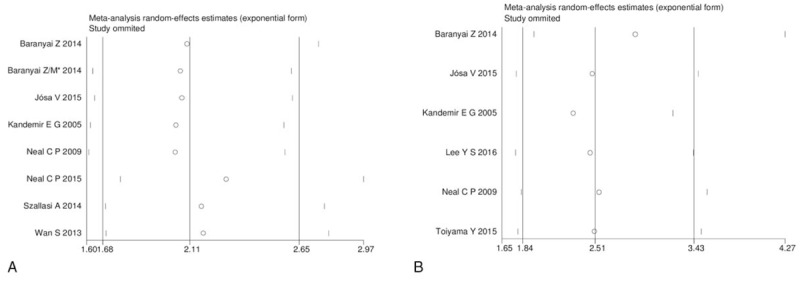
Sensitivity analyses of included studies evaluating hazard ratios of overall survival (A) and disease-free survival (B).

Shorter OS was associated with elevated platelet counts than with normal counts (HR 2.11, 95% CI: 1.68–2.65, I^2^ = 0%) in colorectal cancer^[5,10,15,24,25,27,29]^ (Fig. 2). Elevated platelet counts were also a poor predictor of DFS (HR 2.51, 95% CI: 1.84–3.43, I^2^ = 0%)^[5,10,24–26,28]^ (Fig. 2). In the subgroup analyses, which were used to explore potential heterogeneity among the included studies according to sample size, cut-off values, and the type of colorectal cancer, we consolidated the pooled outcomes. However, the subgroup analyses based on OS demonstrated that there was potential heterogeneity in sample size among the studies. The details of the outcomes of the subgroup analyses based on OS and DFS are presented in Table 4. Moreover, a sensitivity analysis was performed for both OS and DFS among the studies, and the results demonstrated that there no study had a significant influence on the results (Fig. 3). All of the above results suggest that the pooled outcomes were reliable.

**Table 4:**
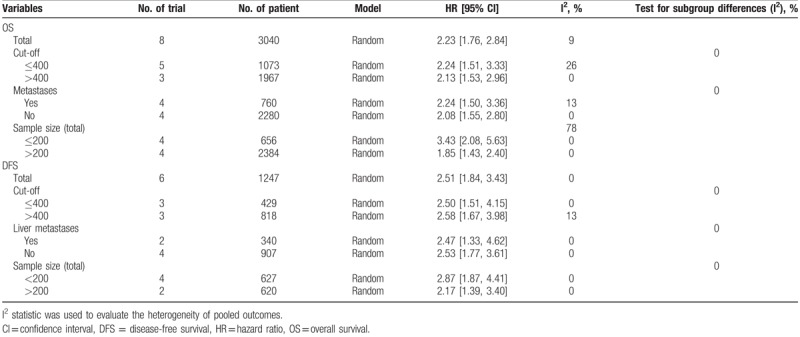
Subgroup analyses’ results.

Interestingly, the subgroup analyses demonstrated that an elevated platelet count was associated with poor OS in both primary colorectal cancer^[5,15,25,29]^ and resectable colorectal liver metastases,^[5,10,24,27]^ with individual HRs that were 2.24 (95% CI: 1.50–3.36, I^2^ = 0%) and 2.08 (95% CI: 1.55–2.80, I^2^ = 13%), respectively. For DFS, an elevated platelet count predicted worse primary colorectal cancer^[5,25,26,28]^ and resectable colorectal liver metastases,^[10,24]^ with individual HRs of 2.53 (95% CI: 1.77–3.61, I^2^ = 0%) and 2.47 (95% CI; 1.33–4.62, I^2^ = 0%), respectively.

## 4 Discussion

In recent decades, it has been observed that thrombocytosis is associated with prognosis in patients with a variety of cancers.^[19,20,30]^ Many studies have demonstrated that elevated platelet counts promote tumor growth, invasion, and metastasis.^[2,4,31–33]^ Although the interaction between the thrombocytosis and cancer has not been clarified, there is sufficient available evidence to propose a mechanism that might potentially underlie this relationship. First, platelets may combine with circulating cancer cells, and the presence of a higher concentration of platelets could more easily lead to the formation of a venous thrombus. Additionally, to some extent, metastatic emboli would form more easily, and this would promote cancer cell implantation.^[34]^ Second, platelets act as “cloaks” for circulating cancer cells by shielding them from the cytotoxic activities of natural killer cells.^[11]^ Platelet-derived growth factor (PDGF) and transforming growth factor β participate in this “shielding process.” Cancer cells that combine with platelets can also act as a “spy” that closely resembles platelets, allowing their hematogenous dissemination via the blood circulation. The “spy” could then adhere to normal tissues or organs by expressing platelet/megakaryocytic gene products.^[5,11]^ Third, platelets might secrete a variety of growth factors and angiogenesis-regulating proteins to promote the generation of tumors and their metastasis. These factors include IL-6, PDGF, platelet factor 4, and vascular endothelial growth factor.^[9,35,36]^ Recently, several studies have also presented data that suggest that the interaction between platelets and cancer is reciprocal and that cancer cells might first simulate platelet activity and production, and then platelets might promote tumor growth, invasion, and metastasis.^[5]^

Based on the results of the above-described studies, many clinical studies have been conducted and many meta-analyses have demonstrated that an elevated platelet count is a negative prognostic predictor of survival in a variety of cancers.^[18–21]^ However, it remains unknown whether elevated platelet counts are associated with worse survive in colorectal cancer patients. For example, in one study that included 630 patients and used a cut-off platelet count value of more than 450 × 10^9^, the authors did not find a significant association between elevated platelet counts and survival.^[16]^ We therefore performed this meta-analysis to clarify this issue.

The results of this meta-analysis demonstrate that in colorectal cancer, shorter OS and DFS were associated with elevated platelet counts than with normal counts. In addition, a subgroup analysis conducted according to sample size, cut-off values, and type of colorectal cancer was performed to consolidate the pooled outcomes. However, in the subgroup analyses based on OS, we found that there was potential heterogeneity among the included studies, possibly because of sample size and the fact that small numbers of patients were included in the groups of included studies, especially the high platelet count group. While it did not appear to matter whether the sample size was more or less than 200, the subgroup analysis demonstrated that an elevated platelet count was associated with worse OS. Interestingly, this meta-analysis demonstrated that both OS and DFS were shorter in patients with elevated platelet counts who had either primary colorectal cancer or resectable colorectal liver metastases. To improve the pooled results, we conducted a sensitivity analysis to validate the credibility of the pooled outcomes. We removed each study one at a time and found that the pooled outcomes were not markedly impacted by any single study. All of the above results strongly indicate that elevated platelet counts may be a reliable predictor of OS and DFS in colorectal cancer patients.

There are several limitations in our study. First, a diversity of cut-off values was used for platelet counts among the different studies included in this meta-analysis. Although the subgroup analysis of platelet counts did not significantly alter our results, the lack of a consistent and precise cut-off value that can be used to combine sensitivity and specificity in a clinical application remains an issue. Second, platelet counts can be influenced by several diseases and drugs, including blood coagulation disorders, blood diseases, splenic disease, and aspirin. Hence, in the future, studies should exclude those factors to more rigorously demonstrate the prognostic value of platelet counts. Third, only 9 studies were included in this analysis, and they were published only in English. We therefore could not exclude publication bias. Fourth, because only 9 retrospective studies were included in the meta-analysis, the underlying heterogeneity among the studies cannot be ignored. However, we performed subgroup and sensitivity analyses to validate the credibility of the pooled outcomes. Finally, small numbers of patients were included in the groups of included studies, especially in the high platelet group, and more studies with larger-scale sample sizes are therefore needed to obtain more reliable results. This is one of the reasons that we have written this article.

## 5 Conclusion

The findings of this meta-analysis suggest that an elevated platelet count is a negative predictor of survival in both primary colorectal cancer and resectable colorectal liver metastases. These data may provide new ideas and evidence for clinical applications aimed at evaluating prognoses in patients with colorectal cancer. A less expensive and simpler method of bio-prediction may therefore be developed in the near future.

## Author contributions

**Conceptualization:** Xu-Dong Rao, Hua Zhang.

**Data curation:** Xu-Dong Rao, Hua Cheng.

**Formal analysis:** Xu-Dong Rao, Hua Zhang, Zheng-Shui Xu, Hua Cheng.

**Methodology:** Zheng-Shui Xu, Wei Shen, Xin-Ping Wang.

**Supervision:** Xu-Dong Rao, Hua Zhang, Zheng-Shui Xu, Hua Cheng, Wei Shen.

**Validation:** Xu-Dong Rao, Hua Zhang.

**Visualization:** Xu-Dong Rao, Zheng-Shui Xu, Hua Cheng.

**Writing – original draft:** Zheng-Shui Xu, Wei Shen, Xin-Ping Wang.

**Writing – review and editing:** Zheng-Shui Xu, Wei Shen, Xin-Ping Wang.

## Supplementary Material

Supplemental Digital Content
