# Supplementary material for: Poor prognostic role of the pretreatment platelet counts in colorectal cancer: A meta-analysis
Source: Medicine (Baltimore). 2018 Jun 18;97(23):e10831. doi: 10.1097/MD.0000000000010831 (PMC5999498; doi:10.1097/MD.0000000000010831)
Supplement: Supplemental Digital Content [file medi-97-e10831-s001.docx]

**Search Strategy in PubMed**

((("Blood Platelets"[Mesh]) OR ((("thrombocytosis"[Title/Abstract]) OR "thrombocythemia"[Title/Abstract]) OR platelet*[Title/Abstract]))) AND((((((((((“colorectal”[Title/Abstract])OR“colorectum”[Title/Abstract]) OR “colon”[Title/Abstract]) OR “Rectum”[Title/Abstract]) OR “Rectal”[Title/Abstract]) OR “large intestine”[Title/Abstract])) AND (((((((adenocarcinoma*[Title/Abstract]) OR tumour*[Title/Abstract]) ORtumor*[Title/Abstract])OR neoplas*[Title/Abstract])ORcarcinoma*[Title/Abstract]) OR cancer*[Title/Abstract]) OR malignant[Title/Abstract]))) OR ((("Colorectal Neoplasms"[Mesh]) OR "Rectal Neoplasms"[Mesh]) OR "Colonic Neoplasms"[Mesh]))

**Search Strategy in EMBASE**

'colon':ab,ti OR colorectal:ab,ti OR colorectum:ab,ti OR rectum:ab,ti OR rectal:ab,ti

OR 'large intestine':ab,ti AND (adenocarcinoma*:ab,ti OR tumour*:ab,ti OR tumor*:ab,ti OR neoplas*:ab,ti OR carcinoma*:ab,ti OR cancer*:ab,ti OR malignant:ab,ti) OR 'rectum tumor'/exp OR 'colorectal tumor'/exp OR 'colon tumor'/exp AND ('thrombocyte'/exp OR thrombocytosis:ti OR thrombocythemia:ti OR platelet*:ti)

**Search Strategy in** **Cochrane Library databases**

#1 "thrombocytosis":ti,ab,kw or "thrombocythemia":ti,ab,kw or platelet*:ti,ab,kw (Word variations have been searched)

#2 MeSH descriptor: [Blood Platelets] explode all trees

#3 #1 or #2

#4 large intestine":ti,ab,kw or "colorectal":ti,ab,kw or "colorectum":ti,ab,kw or "colon":ti,ab,kw or "Rectum":ti,ab,kw (Word variations have been searched) 17442

#5 Rectal":ti,ab,kw (Word variations have been searched)

#6 #4 or #5

#7 adenocarcinoma*:ti,ab,kw or tumour*:ti,ab,kw or tumor*:ti,ab,kw or neoplas*:ti,ab,kw or carcinoma*:ti,ab,kw (Word variations have been searched)

#8 malignant:ti,ab,kw (Word variations have been searched)

#9 #7 or #8

#10 #6 and #9

#11 MeSH descriptor: [Colorectal Neoplasms] explode all trees

#12 MeSH descriptor: [Rectal Neoplasms] explode all trees

#13 MeSH descriptor: [Colonic Neoplasms] explode all trees

#14 #10 or #11 or #12 or #13

#15 #14 and #3
